# Effect of Reversible Addition-Fragmentation Transfer Emulsion Styrene Butadiene Rubber (RAFT ESBR) on the Properties of Carbon Black-Filled Compounds

**DOI:** 10.3390/polym12040933

**Published:** 2020-04-17

**Authors:** Kiwon Hwang, Hyunsung Mun, Wonho Kim

**Affiliations:** Department of Polymer Science & Chemical Engineering, Pusan National University, Busan 609-735, Korea; kiwon8348@gmail.com (K.H.); ansehdwns10@gmail.com (H.M.)

**Keywords:** RAFT polymerization, carbon black-filled compound, emulsion styrene-butadiene rubber, crosslink density, vulcanizate structure

## Abstract

Tread is an important component that directly affects the performance of passenger car radial (PCR) tires. Styrene-butadiene rubber (SBR) is mainly used for tire tread and it includes solution styrene-butadiene rubber (SSBR) and emulsion styrene-butadiene rubber (ESBR). Although SSBR is mainly used, the manufacturing process for SSBR is more challenging than ESBR, which is environmentally friendly, but has the disadvantage of a broad molecular weight distribution. To overcome this, a reversible addition-fragmentation radical transfer (RAFT) polymerization technique is used in ESBR polymerization. An environmentally friendly RAFT ESBR with a narrow dispersity can be polymerized. Here, carbon black-filled compounds were manufactured while using RAFT ESBR, and their properties were compared to ESBR. The analysis showed a low crosslink density of RAFT ESBR, due to the high polysulfide crosslink structure. We manufactured a carbon black-filled compound with the same crosslink density and structure as the ESBR carbon black-filled compound, and the effect of the dispersity of the base polymer was investigated. RAFT ESBR showed 9% better abrasion resistance and 29% better fuel efficiency than ESBR, according to the analysis of the data. The narrow dispersity can reduce energy loss and positively influence the abrasion resistance and fuel efficiency.

## 1. Introduction

Tires have a complex structure that consists of more than 10 components, such as tread, belts, carcass, sidewall, and inner liner, etc., among which treads are used to directly affect tire performance. The rubber compound must satisfy fuel efficiency (rolling resistance), traction performance, and abrasion resistance, etc., simultaneously. Among these properties, traction performance can be used to determine the driving safety, and tire treads directly contacting with the road surfaces have a great influence [1,2,3]. Therefore, several research studies have focused on applying styrene-butadiene rubber (SBR), which has excellent traction ability, to the tire tread rubber compound for passenger car radials (PCRs) [4,5,6].

SBR is classified into emulsion styrene-butadiene rubber (ESBR) and solution styrene-butadiene rubber (SSBR), depending on the polymerization method. The polymerization of ESBR uses water as a solvent and SSBR uses an organic solvent, and they are polymerized by different mechanisms. The advantages of SSBR include microstructure control, chain-end functionalization, and narrow dispersity, but it has the disadvantages of having a high manufacturing cost and requiring the use of organic solvents. When compared to SSBR, ESBR is more environmentally friendly and it is easier to obtain a high molecular weight polymer, which has advantages in terms of mechanical properties [7]. However, microstructure and/or chain-end functionalization is difficult to control for ESBR, and polymerization is carried out through free radical polymerization; therefore, the dispersity is broad [8]. In general, tire properties are highly influenced by the macrostructure (molecular weight and dispersity, etc.) and microstructure (chain architecture, vinyl content, branch of the polymer chain, etc.) of the polymer that is used [9]. In the case of ESBR, the dispersity is broad and chain branches are formed due to the radical polymerization characteristics. Consequently, the hysteresis loss increases, which causes unfavorable results compared to SSBR in terms of the dynamic viscoelastic properties [10].

As the dynamic viscoelastic properties of tread compounds are directly related to the fuel efficiency of tires, the broad dispersity of ESBR needs to be improved. In general, a method of effectively narrowing the broad dispersity of polymers is to apply the reversible deactivation radical polymerization (RDRP) technique. There are three major types of living radical polymerizations, depending on the mechanism: atom transfer radical polymerization (ATRP) [11], nitroxide-mediate radical polymerization (NMP) [12], and reversible addition-fragmentation radical transfer (RAFT) polymerization [13]. Among these polymerization techniques, RAFT polymerization can be applied to various reaction conditions and it can be performed in the conventional free radical polymerization set-up. In addition, the polymerization conditions (for example, solvents, temperature, etc.) are environmentally friendly when compared to other living radical polymerizations, as there are no metal ligands or toxic solvents [14,15,16,17,18,19].

**Figure 1:**
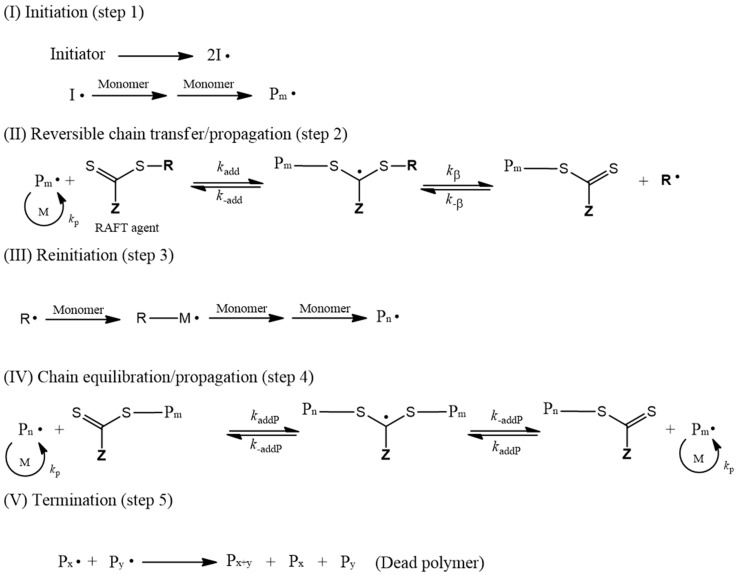
Scheme of reversible addition-fragmentation radical transfer (RAFT) polymerization.

Usually, RAFT polymerization uses thiocarbonylthio compounds having a generic formula R–S–(C=S)–Z as a chain transfer agent (RAFT agent) [20,21,22]. The RAFT agents are highly active chain transfer agents that allow for a dynamic equilibrium between propagating radicals and dormant species through fast degenerative chain transfer reactions [23,24]. This rapid dynamic equilibrium provides an equal probability of the polymer chains growing uniformly, and making the polymerized polymers have a narrow dispersity [25] (Figure 1). Furthermore, RAFT polymerization is most suitable for the polymers with large molecular weights and it can be applied to emulsion polymerization. Recently, research on RAFT emulsion polymerization while using the RAFT polymerization technique has been extensively studied [26,27,28,29,30]

However, unlike solution or bulk polymerization, it has been reported that the application of the RAFT polymerization technique to emulsion polymerization causes some problems such as loss of molecular weight control, difficulties of coagulum formation, and phase separation [31,32,33,34,35]. In the case of RAFT emulsion polymerization, polymerization occurs after the RAFT agent is diffused into the micelle particles due to the polymerization characteristics. Therefore, the above problems occur, depending on the structure and solubility of the RAFT agent. When the RAFT agent has a monomer-soluble structure, the RAFT agent has a good affinity with the monomer droplet. Thus, the diffusion rate of the RAFT agent into the micelle particles is low, such that the polymerization proceeds with the same mechanism as the conventional emulsion polymerization and it cannot mediate the polymerization reaction. In addition, when the RAFT agent has a water-soluble structure, a chain transfer reaction of the RAFT agent occurs in the water phase. Consequently, it takes a long time to form oligomeric radicals that can enter the micelle particles and act as a retardant of polymerization [36].

Research on the polymerization of RAFT ESBR using this RAFT emulsion polymerization technique has been recently reported [37]. In early RAFT ESRB polymerization, other RAFT agents, except *S,S*-dibenzyl trithiocarbonate (DBTC), formed a gel and it could not effectively obtain a high molecular weight due to the problem of the slow diffusion rate of the RAFT agent into emulsion particles [38]. Based on the reference 37 and 38, our researchers also carried out the polymerization of RAFT ESBR while using RAFT agents of various structures for the selection of appropriate RAFT agent. When the RAFT agent has hydrophilic functional groups (i.e., *S*-(thiobenzoyl) thioglycolic acid), the chain transfer radicals that formed by fragmentation were present in this water phase due to its water-soluble structure. Additionally, in the case of SBR polymerization, butadiene having a low propagation rate was used as a monomer. Therefore, when the RAFT agent with hydrophilic functional groups was used for ESBR polymerization, chain transfer radical acted as retardants, and polymerization conversion was low due to the water-soluble structure of RAFT agent and low propagation rate of butadiene monomer. In addition, when the RAFT agent has monomer-soluble structure (i.e., benzyl-benzodithioate or 2-phenyl-2-propyl-benzodithioate), gel was formed during the polymerization due to the slow diffusion rate of chain transfer radical into micelle particles. Accordingly, according to our research, *S,S*-dibenzyl trithiocarbonate (DBTC) showed a proper capability of dispersity control for the polymerization of high molecular weight RAFT ESBR.

However, recently, RAFT ESBR polymerization studies reported solving these kinds of problem, and Yu et al. attempted to solve the problem of the slow diffusion rate of RAFT agents into micelle particles while using a mini emulsion polymerization technique [39]. In addition, Mun et al. reported the results of polymerization of RAFT ESBR using DBTC as a RAFT agent, having a similar molecular weight as conventional ESBR, but with a narrower dispersity. The unfilled compounds were manufactured using the polymerized RAFT ESBR, and the properties were compared with conventional ESBR compounds. It was confirmed that RAFT ESBR has excellent abrasion resistance and fuel efficiency, despite the low crosslink density [40]. According to Mun et al., the RAFT agent used as a chain transfer agent formed a polysulfide structure by reacting with sulfur, resulting in lower crosslink density of the compounds, or shortened SBR chain length, by reacting with a silane coupling agent. Consequently, unlike the unfilled compound, RAFT ESBR silica filled compounds showed unfavorable abrasion resistance and fuel efficiency as compared to the conventional ESBR. In the case of RAFT ESBR carbon black-filled compounds (which do not use the silane coupling agent), despite the low crosslink density due to the formation of polysulfide structure, RAFT ESBR showed similar abrasion resistance and excellent fuel efficiency due to having the same molecular weight and narrow dispersity [41]. However, previous studies did not analyze the crosslink structure of carbon black-filled compounds or evaluate the properties in the compounds with the same crosslink density.

Therefore, in this study, the crosslink structure of RAFT ESBR carbon black-filled compounds was analyzed, and a carbon black-filled RAFT ESBR compound with the same crosslink density as the carbon black filled ESBR compound was manufactured in order to investigate the effect of the narrow dispersity of RAFT ESBR on the mechanical and dynamic viscoelastic properties. Accordingly, after the polymerization of RAFT ESBR having a high molecular weight and narrow dispersity, the RAFT ESBR carbon black-filled compound was manufactured and the polysulfide structure was broken by applying a mixture of propane-2-thiol and hexylamine. A swelling test was also carried out to quantitatively analyze the crosslink structure of the vulcanizates. Based on the crosslink density analysis results, the properties of the RAFT ESBR carbon black-filled compound, for which the crosslink density was controlled by cure system regulation, were compared with the conventional ESBR carbon black-filled compound. Finally, the effect of narrow dispersity of the RAFT ESBR on the mechanical and dynamic viscoelastic properties of carbon black-filled compounds was investigated.

## 2. Materials and Methods

### 2.1. Materials for ESBR Polymerization

For the polymerization of ESBR and RAFT ESBR, styrene (99.5%) was purchased from SAMCHUN Chemicals, Korea, and 1,3-butadiene, a surfactant (fatty soap, rosin soap), micelle stabilizer (KOH), and *p*-methane hydroperoxide (as an initiator) were supplied by Kumho Petrochemical Co. Ltd. (KKPC, Seoul, Korea) and then used without further purification. Tert-Dodecyl mercaptan (TDDM) as a chain transfer agent, *S,S*-dibenzyl trithiocarbonate (DBTC) (97%) as a RAFT agent, sodium hydrosulfite (SHS) as a reducing agent, sodium formaldehyde sulfoxylate (SFS, 86%) as a catalyst, ferrous sulfate (FES, 99%), ethylenediaminetetraacetic acid (EDTA, 99%), and diethylhydroxylamine (DEHA, 98%) as a shortstop agent, were purchased from Merck, Kenilworth, NJ, USA. As a coagulant, NaCl and H_2_SO_4_ (1 mol/L) were purchased from Daejung, Korea.

### 2.2. Materials for Manufacturing a Carbon Black-Filled Compound and Evaluating Crosslink Density

The ESBR and RAFT ESBR were used after coagulating latex with NaCl and H_2_SO_4_. Carbon black N330 (OCI, Korea) was used as a filler when manufacturing the carbon black-filled compound. Zinc oxide (ZnO) and stearic acid (CH_3_(CH_2_)_16_COOH) as additives, *N*-(1,3-dimethybutyl)-*N’*-phenyl-phenylenediamine (6PPD) as an antioxidant, sulfur, *N*-cyclohexyl benzothiazyl sulfenamide (CBS), and diphenyl guanidine (DPG) as vulcanization agents, were purchased from Merck, Kenilworth, NJ, USA.

Tetrahydrofuran (THF, Daejung, Korea) and *n*-hexane (Daejung, Busan, Korea) were used in order to remove organics from the vulcanizates prior to the swelling experiments. Toluene (Daejung, Korea) was used to confirm the crosslink density. Piperidine (Daejung, Korea), propane-2-thiol (Acros Organics, Waltham, MA, USA), and n-heptane (Samchun, Seoul, Korea) were used to destroy the vulcanizates structure.

### 2.3. Polymerization of ESBR

The ESBR and RAFT ESBR were polymerized by low-temperature emulsion polymerization through the following process. In a high-pressure stainless-steel stirrer reactor (2 L), styrene, water, rosin soap, fatty soap, electrolyte, sodium hydrosulfite, activate solution, and chain transfer agent were charged and purged with nitrogen gas. The TDDM was used for ESBR and DBTC was used for RAFT EBSR as chain transfer agents. TDDM, which is an irreversible chain transfer agent, can lose its activity to regulate the dispersity of a polymer after a transfer reaction, whereas DBTC, a reversible chain transfer agent, can reversibly transfer the active radical to a dormant species and make it reactive. Subsequently, the initiator was added and 1,3-butadiene (measured in a small chamber) was injected using a nitrogen pressure of 3-bar through a gas line connected to the reactor. Polymerization was carried out while maintaining the temperature of the reactor at 9 °C. Table 1 lists a detailed polymerization recipe.

### 2.4. Conversion Measurement and Latex Coagulation

During the polymerization reaction, ESBR was sampled at 2 h intervals and RAFT ESBR, which had a relatively long polymerization time, was sampled at 4 h intervals. The conversion was calculated by measuring the total solids content of the latex that was sampled every interval while using a moisture dryer (MB45, OHAUS, Parsippany, NJ, USA). The reaction was terminated by injecting a shortstop agent when the conversion reached 65%.

NaCl was added up to 15% of the amount of rubber in the polymerized latex and the solution was slowly coagulated by dropping in an aqueous sulfuric acid solution. Coagulated ESBR and RAFT ESBR were washed three times with distilled water and then dried in a circulation hot air-drying oven at 55 °C for 24 h.

### 2.5. Characterization of ESBR and RAFT ESBR Raw Polymers

The contents of styrene, butadiene, and vinyl structure in the polymerized polymer were determined while using nuclear magnetic resonance spectrometer (^1^H-NMR; Varian, Unity Plus 300 spectrometer, Garden State Scientific, Morristown, NJ, USA).

The molecular weight and dispersity were determined by GPC (gel permeation chromatography, DGU 20A 3R, Shimazu, Kyoto, Japan) using a solvent delivery unit, refractive index detector, and styragel column (High Temperature [HT] 6E, 10 μm, Φ 7.8 mm × 6300 mm; High Molecular Weight [HMW] 7, 15–20 μm, Φ 7.8 mm × 300 mm; HMW 6E, 15–20 μm, Φ 7.8 mm × 300 mm). The GPC calibration curves were prepared using a polystyrene standard.

Mooney viscosity was measured using a Mooney viscometer (Vulchem IND Co., Korea), which is a type of rotatory viscometer. After preheating for 1 min. according to ASTM D 1646 conditions at 100 °C, the large disk (38.10 ± 0.05 mm, thickness 5.5 ± 0.05 mm) was measured by operating at 2 rpm for 4 min.

### 2.6. Manufacture of Compounds and Vulcanizates

The carbon black-filled compounds were manufactured by applying the formulation shown in Table 2 to quantitatively analyze the crosslink structure of the manufactured vulcanizates and determine the effect of crosslink density of vulcanizates on mechanical and dynamic properties. The carbon black filler was variably added to clearly distinguish between total crosslink density and chemical crosslink density through the Kraus plot. The compound was manufactured by applying the formulation shown in Table 3 to increase the crosslink density of carbon black-filled compound using RAFT ESBR.

The compounds were manufactured using an internal Kneader (300 cc, MIRAESI Company, Incheon, Korea), and the fill factor was set to 0.7 and proceeded in two steps. In the first stage, kneading commenced at 110 °C, and the carbon black masterbatch was kneaded for 12 min. at the dump temperature of 150–155 °C. After the first stage of kneading, sulfur and cure accelerator *N*-*tert*-*butyl*-2-benzothiazyl sulfonamide (TBBS) were added and kneaded at 50 °C for 2 min. during the second stage. Table 4 lists the detailed procedure.

### 2.7. Experimental Methods for Carbon Black-Filled Compounds

All of the experiments were repeated 3–4 times and the difference in each average value was insignificant. For this reason, error bars are not included with the graphs of the data.

#### 2.7.1. Cure Characteristics

The cure characteristics of the manufactured carbon black-filled compounds were measured using a moving die rheometer (RLR-3; rotorless rheometer, Toyoseiki, Nagano, Japan) for 20 min. at 160 °C with an oscillation angle of ±1°. Through this experiment, it is possible to determine the minimum and maximum torque values and the optimum vulcanization time (t90). Subsequently, vulcanizates were manufactured while using a press with optimum vulcanization time at 160 °C.

#### 2.7.2. Analysis of vulcanizate Structure (Swelling Test)

The crosslinked structure was evaluated by measuring the crosslink density. The vulcanizates were cut into 10 mm (length) × 10 mm (width) × 2 mm (thickness) sizes and then stored for 48 h in 30 mL of THF (tetrahydrofuran) and 30 mL of *n*-hexane, respectively, to remove organic additives. After drying the samples for one day at room temperature to remove the organic additives, the samples were weighed, and were then swollen in toluene for 24 h and weighed again. Finally, the total crosslink density was calculated using the measured weight and the Flory–Rehner Equation (1), below.

(1) $$v=\frac{1}{2M_{c}}=-\frac{\ln\left(1-V_{1}\right)+V_{1}+\chi V_{1}^{2}}{2\rho_{r}V_{0}\left(V_{1}^{\frac{1}{3}}-\frac{V_{1}}{2}\right)}$$

Here, *ν* is the crosslink density (mol/g), *M_C_* is the average molecular weight between crosslink points (g/mol), *V*_1_ is the volume fraction of rubber in the swollen gel at equilibrium, *V*_0_ is the molar volume of solvent (cm^3^/mol), *ρ_r_* is the density of the rubber sample (g/cm^3^), and *χ* is the polymer–solvent interaction parameter.

For further analysis of the crosslink structure, a mixture of n-heptane (50 mL), propane-2-thiol (0.4 M), and hexylamine (0.4 M) was prepared. The samples, after removing the organic additives, were stored at room temperature for 24 h in the mixture solution. Subsequently, the sample was removed and dried, and then swollen in toluene for 24 h. Di- and mono-crosslinks were measured using the difference in the sample weight before and after swelling [42,43].

The total crosslink density (chemical crosslink density + filler-rubber interaction) and chemical crosslink of the unfilled compound were obtained using the Flory–Rehner Equation (1) and Kraus Equation (2). The degree of filler-rubber interaction was determined by calculating the difference in the densities [44,45,46,47].

(2) $$\frac{v_{r0}}{v_{r}}=1-m\left(\frac{\varphi }{1-\varphi }\right)$$

Here, *φ* is the volume fraction of filler, *ν*_*r*0_ is the volume fraction of unfilled rubber in the swollen gel, and *ν*_r_ is the volume fraction of rubber in the swollen gel.

#### 2.7.3. Mechanical Properties

Dumbbell-shaped specimens of 100 mm (length) × 25 mm (width) × 2 mm (thickness) specifications were prepared according to ASTM D412. The prepared specimens were measured for mechanical properties (tensile strength, elongation at break, and tensile modulus) of vulcanizates while using a universal testing machine (UTM, Model; KSU-05M-C, KSU Co., Korea).

#### 2.7.4. Abrasion Loss

Cylindrical specimens of 16 mm diameter and 8 mm thickness were prepared according to DIN 53516 and the initial mass of the specimens were measured. Subsequently, the mass of the abraded specimen was measured to determine the abrasion loss after grinding the specimens for 40 m at 40 rpm using a Deutsche Industrie Normen (DIN) abrasion tester.

#### 2.7.5. Dynamic Viscoelastic Properties

Specimens of 15.0 mm (length) × 5.0 mm (width) × 2.0 mm (thickness) were prepared for measuring the dynamic viscoelastic properties of vulcanizates. The glass transition temperature and dynamic viscoelastic properties of the vulcanizates were measured while using a dynamic mechanical thermal analyzer (DMTA, EPLEXOR 500N, GABO, München, Germany) in tension mode with an amplitude of 30 μm, frequency of 10 Hz, and temperature range of −80 °C to 80 °C.

## 3. Results and Discussion

### 3.1. Characterization of ESBR and RAFT ESBR

**Figure 2:**
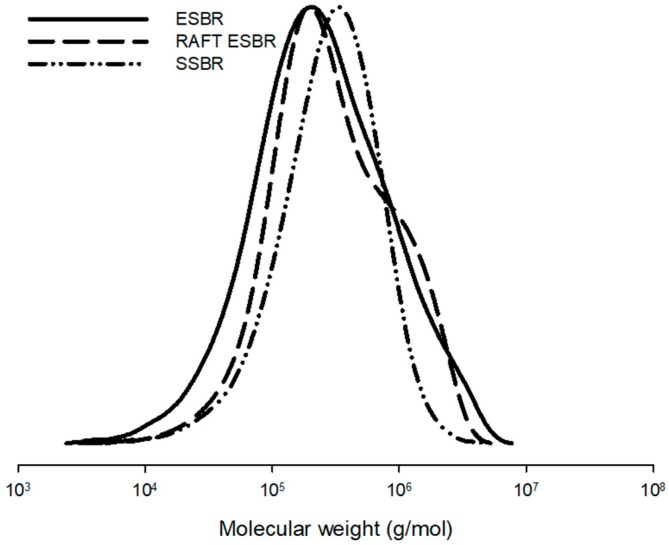
Gel permeation chromatography (GPC) curves of emulsion styrene butadiene rubber (ESBR), reversible addition-fragmentation radical transfer ESBR (RAFT ESBR), and solution styrene butadiene rubber (SSBR).

The molecular weights of ESBR and RAFT ESBR were determined using GPC. For ESBR, the weight average molecular weight (M_w_) was 549,000 and the dispersity was 4.1. In contrast, RAFT ESBR had a weight average molecular weight (M_w_) of 550,000 and a dispersity of 3.1, confirming that it had a narrower polydispersity than ESBR. The Mooney viscosities of ESBR and RAFT ESBR were 52 and 61, respectively. In the GPC measurement graph (Figure 2), the RAFT ESBR with a narrow dispersity had fewer “polymer chains of low molecular weight” than that of ESBR. If fewer of these “polymer chains of low molecular weight” were formed, then the zero-shear viscosity value was higher, and the polymer would have exhibited a greater Newtonian behavior than the polymer with the broad dispersity, resulting in less shear thinning behavior during deformation [48,49,50,51]. Consequently, the elastic characteristics of the RAFT ESBR polymer appeared to be more Newtonian than ESBR, which corresponds well with the higher Mooney viscosity of the RAFT ESBR than the ESBR. 

**Figure 3:**
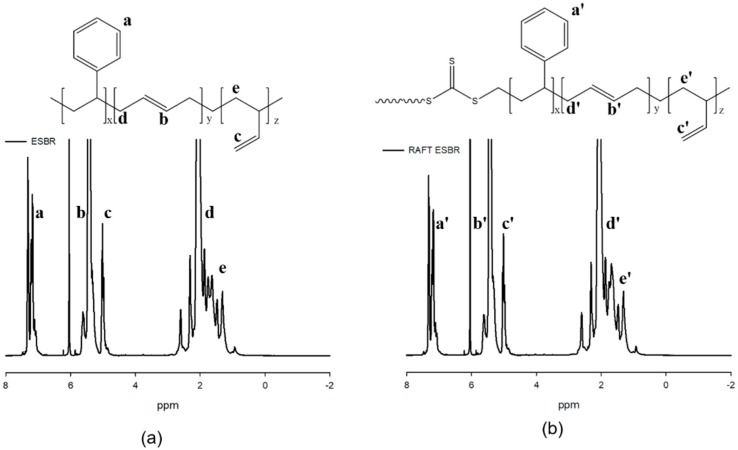
Nuclear magnetic resonance spectrometer (NMR) curves of (**a**) emulsion styrene butadiene rubber (ESBR) and (**b**) reversible addition-fragmentation radical transfer ESBR (RAFT ESBR) raw polymers.

In addition, the microstructure of the polymers was confirmed using ^1^H-NMR. In the SBR structure, the 1,2-vinyl group showed a peak at 4.6–5.0 ppm, at 5.0–5.75 ppm for 1,4-addition of butadiene, and 6.7–7.3 ppm for styrene. Among the 1,4 additions of butadiene, *cis*-1,4 addition showed peaks at 5.3–5.4 ppm and *trans*-1,4 addition at 5.4–5.5 ppm. The microstructure compositions of the polymerized ESBR and RAFT ESBR were calculated while considering the molecular weight of the monomer and the area of each peak. The styrene contents and butadiene contents in the ESBR and RAFT ESBR were 23.5% and 76.5%, respectively, and the vinyl contents were 20.7% and 21.1%, respectively. It was confirmed that the two polymers had a similar microstructure, according to the ^1^H-NMR measurement results. Figure 2 and Figure 3, and Table 5 show detailed GPC and ^1^H-NMR results.

### 3.2. Properties of ESBR and RAFT ESBR Carbon Black-Filled Compounds

#### 3.2.1. Analysis of Curing Characteristics and Crosslink Structure of ESBR and RAFT ESBR Carbon Black-Filled Compounds 

**Figure 4:**
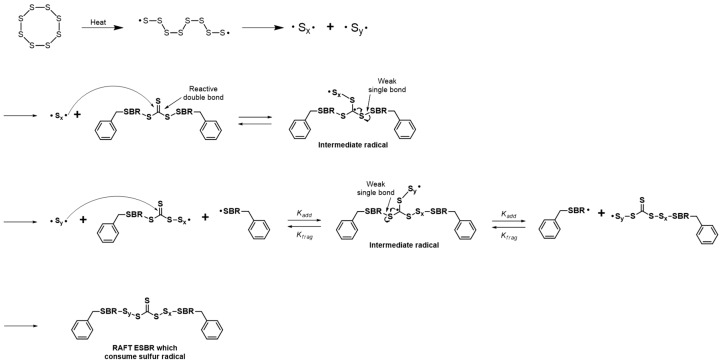
Suggested mechanism of the reaction between reversible addition-fragmentation radical transfer emulsion styrene butadiene rubber (RAFT ESBR) and sulfur.

**Figure 5:**
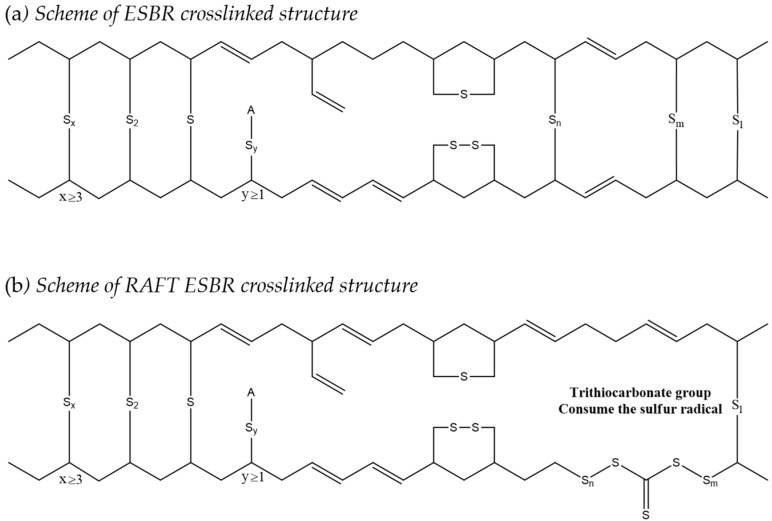
Crosslinked structure schemes of (**a**) emulsion styrene butadiene rubber (ESBR) and (**b**) reversible addition-fragmentation radical transfer ESBR (RAFT ESBR) vulcanizates.

**Figure 6:**
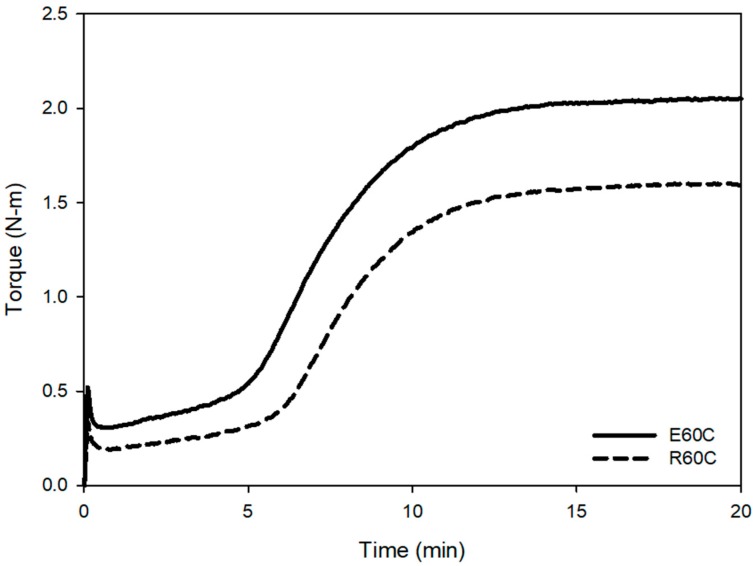
Cure characteristics of emulsion styrene butadiene rubber (ESBR) and reversible addition-fragmentation radical transfer (RAFT) ESBR carbon black-filled compounds.

The main difference in the structure of RAFT ESBR and ESBR is the presence of trithiocarbonate groups in the chain. In the case of RAFT ESBR, trithiocarbonate groups exist between styrene-butadiene backbone chains due to DBTC, a RAFT agent that is used during RAFT polymerization. As shown in Figure 4 and Figure 5, during addition-fragmentation of the DBTC RAFT agent, the trithiocarbonate groups consumed some of the thermally generated sulfur –S_8_– radicals and formed different crosslinked structures when compared to conventional ESBR [40]. Accordingly, as shown in Figure 6, the results of the moving die rheometer (MDR) measurement showed that the RAFT ESBR exhibited a lower value than the ESBR compound when comparing (T_max_ − T_min_) results, which are closely influenced by the crosslink density. These results may be due to the high formation of a polysulfide crosslinked structure [52]. 

**Figure 7:**
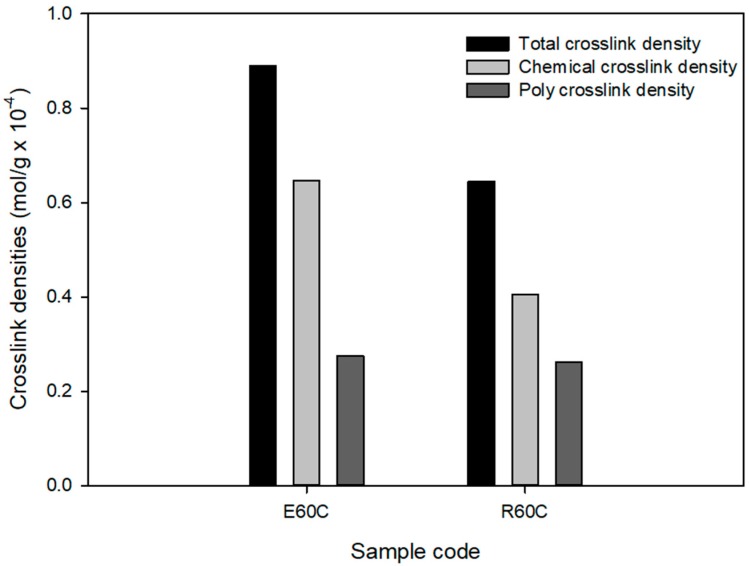
Crosslink densities of vulcanizates of emulsion styrene butadiene rubber (ESBR) and reversible addition-fragmentation radical transfer (RAFT) ESBR carbon black-filled compounds.

The swelling test was used to measure the crosslink densities, and Figure 7 and Table 6 show these results. The total crosslink density was 0.8888 × 10^−4^ mol/g for ESBR and 0.6449 × 10^−4^ mol/g for RAFT ESBR. Based on the MDR results, a lower crosslink density value for RAFT ESBR is expected, and the RAFT ESBR showed a higher ratio of polysulfide crosslink density than ESBR due to the trithiocarbonate group. Additionally, the chemical crosslink and filler-rubber interaction of vulcanizates were separated while using a Kraus plot and the Flory–Rehner equation. In contrast to the results of silica-filled RAFT ESBR compounds [41], carbon black-filled RAFT ESBR compounds formed a polysulfide crosslink while maintaining a similar filler-rubber interaction, because there was no silane agent reducing the molecular weight of the polymers. Accordingly, we confirmed that the chemical crosslink density was significantly reduced in RAFT ESBR carbon black-filled compounds. These results confirmed that, in the case of carbon black-filled RAFT ESBR compounds that do not use a silane agent, the molecular weight does not decrease, and trithiocarbonate groups in the polymer chain simply consume sulfur to form a different crosslinked structure with ESBR. In addition, Mooney viscosity measurement results using the manufactured RAFT ESBR compounds showed a higher Mooney viscosity value than ESRB due to the narrow dispersity of RAFT ESBR.

#### 3.2.2. Dynamic Viscoelastic Properties of ESBR and RAFT ESBR Carbon Black-Filled Compounds

**Figure 8:**
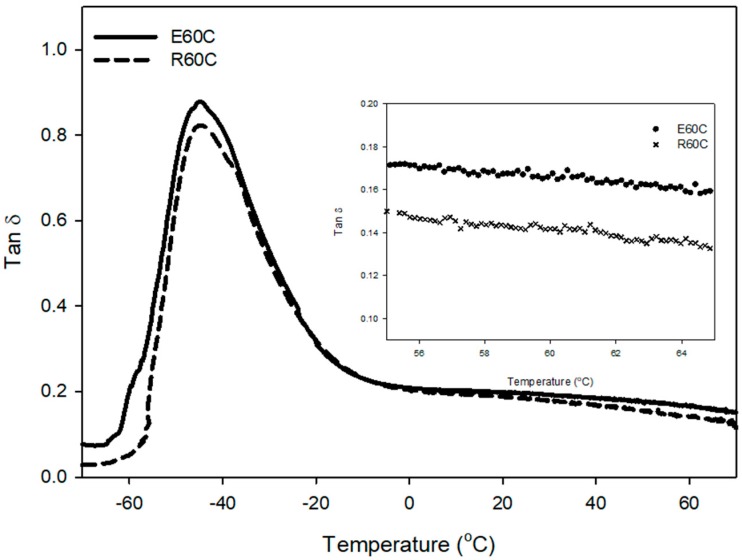
Temperature-dependent tan δ of vulcanizates of emulsion styrene butadiene rubber (ESBR) and reversible addition-fragmentation radical transfer (RAFT) ESBR carbon black-filled compounds.

The results of dynamic viscoelastic properties showed that the temperature range at the glass transition zone of RAFT ESBR is narrower, and the tan δ peak at *T*_g_ is also lower than that of ESBR, as shown in Figure 8 and Table 7. Generally, the dynamic viscoelastic properties of rubber compounds are influenced by the viscoelastic behavior of fillers and polymers. At the low temperatures or near *T*_g_, molecular motion occurring by micro-brown motion of polymers is the main cause of energy dissipation, because filler behavior is restricted in the polymer network [53]. Consequently, the RAFT ESBR that has narrower dispersity and a more uniform polymer chain length showed lower energy dissipation of the polymer and tan δ peak near *T*_g_ than ESRB. Furthermore, the values of tan δ at 0 °C, representative of wet traction, are strongly influenced by the glass transition temperature (*T*_g_) [54], so E60C and R60C compounds with similar *T*_g_ values showed similar values of tan δ at 0 °C. The value of tan δ at 60 °C, representative of rolling resistance, is generally low when the compound has a high crosslink density [55]; however, RAFT ESBR showed a better rolling resistance value, despite the low crosslink density. This confirmed that the number of “polymer chains of low molecular weight” of RAFT ESBR were less than those of ESBR due to the narrow dispersity of the raw polymer. 

#### 3.2.3. Mechanical Properties and Abrasion Resistance of ESBR and RAFT ESBR Carbon Black-Filled Compounds

**Figure 9:**
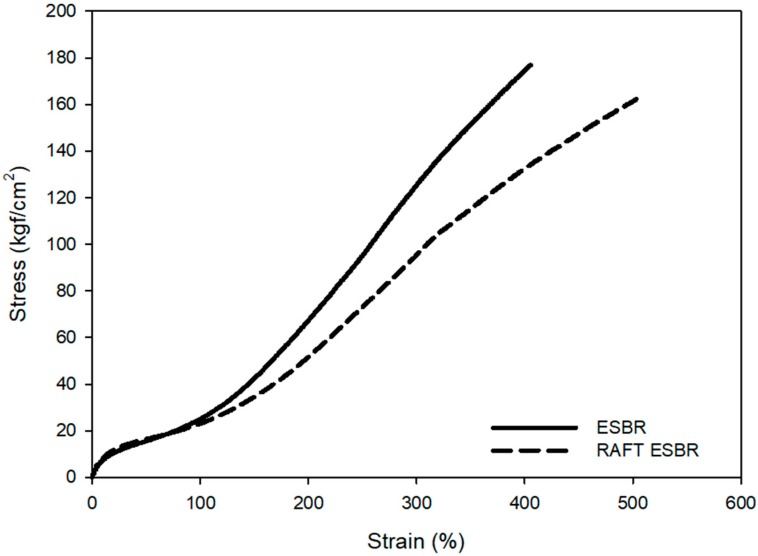
Mechanical properties of vulcanizates of emulsion styrene butadiene rubber (ESBR) and reversible addition-fragmentation radical transfer (RAFT) ESBR carbon black-filled compounds.

In general, the mechanical properties are closely related to the total crosslink density of the compounds [56]. The major difference is the presence or absence of trithicarbonate groups in the SBR polymer chain. Sulfur forms more polysulfide structures than mono or di sulfide structures due to the trithicarbonate groups in the chain. ESBR with a high crosslink density showed higher M_100%_ and M_300%_ than RAFT ESBR, as shown in Figure 9 and Table 8. In addition, RAFT ESBR, which has more polysulfide crosslinked structures, had a more flexible crosslinked structure than ESBR, resulting in higher elongation-at-break values. However, the abrasion resistance results showed that RAFT ESRB had a similar abrasion resistance to ESBR, despite the low crosslink density. Various factors influenced abrasion resistance, and energy loss due to friction with the contacting surface is also one of the important factors affecting the abrasion resistance [57,58]. The RAFT ESBR exhibited a lower energy dissipation than the ESBR because of its lower dispersity, as shown in the results of the dynamic viscoelastic properties. Accordingly, RAFT ESBR showed similar abrasion resistance to ESBR, despite the low crosslink density. 

### 3.3. ESBR and RAFT ESBR Carbon Black-Filled Compounds with Controlled Cure System 

#### 3.3.1. Analysis of Curing Characteristics and Crosslink Structure of Carbon Black-Filled Compounds with a Controlled Cure System

**Figure 10:**
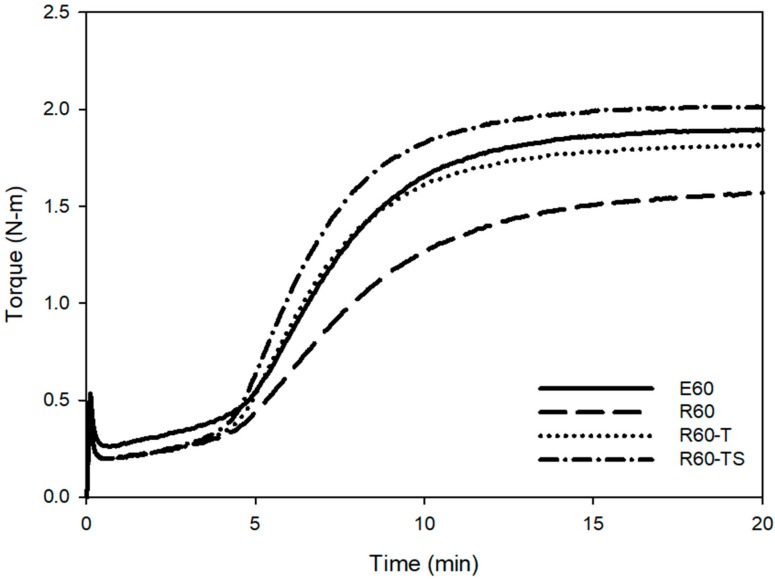
Cure characteristics of carbon black-filled compounds with a controlled cure system.

**Figure 11:**
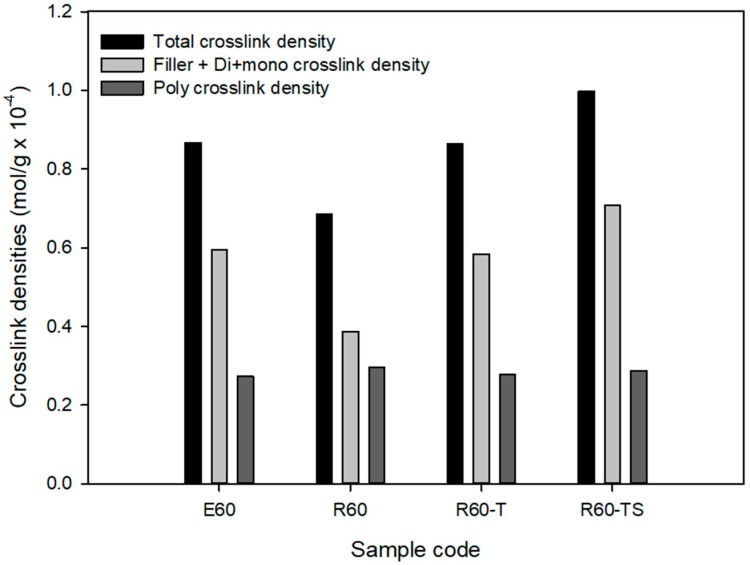
Crosslink densities of vulcanizates of carbon black-filled compound with a controlled cure system.

Through the previous experiments, we confirmed that the low crosslink density of the RAFT ESBR compound adversely affected the mechanical properties when compared to the ESBR compound. It is believed that the trithiocarbonate groups in the RAFT ESBR chains react with sulfur to form polysulfide structures. Compounds with an increased amount of the accelerator and sulfur were manufactured and evaluated to modify the polysulfide crosslink structure of the RAFT ESBR compounds into di- and mono-sulfide crosslinks (as shown in Table 3). As a result of cure characteristics, the R60-T compound in which TBBS, a crosslink accelerator, was increased from 1.0 phr to 1.3 phr showed a similar cure curve to that of E60 compound, as presented in Figure 10. The R60-TS compound with an increased sulfur and TBBS content showed a higher cure curve than the E60 compound. This higher cure curve occurs because the crosslink density was increased due to the additions of the crosslink accelerator and sulfur, as confirmed through crosslink density and crosslink structure analyses. The crosslink density measurements showed similar values of 0.8672 × 10^−4^ mol/g and 0.8630 × 10^−4^ mol/g for the E60 compound and the R60-T compound, as shown in Figure 11 and Table 9, respectively, with an increased amount of accelerator. The use of crosslink structure analysis also confirmed that similar polysulfide crosslinks were formed. As a result of the crosslink density and structure analyses, we concluded that the trithiocarbonate group of RAFT ESBR consumed sulfur through a competition reaction with a crosslink accelerator, and that ESBR and RAFT ESBR compounds having a similar crosslink structure could be manufactured by increasing the crosslink accelerator. The Mooney viscosity measurements of the manufactured compounds indicated a higher Mooney viscosity for the RAFT ESBR compounds with narrow dispersity than the ESRB compounds. In addition, the R60-T and R60-TS compounds that were controlled with a crosslink accelerator showed a decrease in Mooney viscosity, indicating that TBBS that was used as a crosslink accelerator reduced the viscosity in the compound.

#### 3.3.2. Dynamic Viscoelastic Properties of ESBR and RAFT ESBR Carbon Black-Filled Compounds with a Controlled Cure System

**Figure 12:**
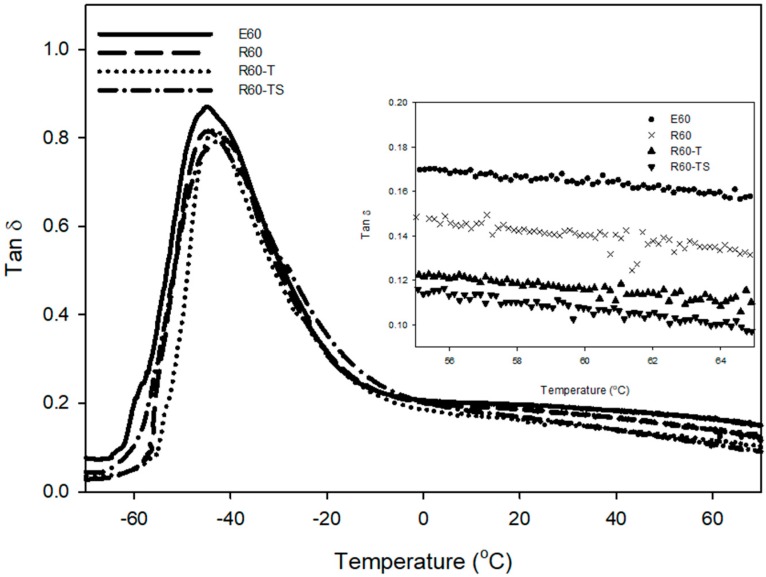
Temperature-dependent tan δ of vulcanizates of emulsion styrene butadiene rubber (ESBR) and reversible addition-fragmentation radical transfer (RAFT) ESBR carbon black-filled compounds with a controlled cure system.

Compounds manufactured using RAFT ESBR have lower tan δ peak values at *T*_g_ than the compound manufactured using ESBR, as shown in Figure 12 and Table 10. This result appears to be due to the low energy dissipation of the polymer because the RAFT ESBR has a narrow dispersity. In addition, tan δ at 60 °C, which was representative of rolling resistance, was lower than that of ESBR due to the narrow dispersity of RAFT ESBR compounds, and R60-TS with the highest crosslink density showed the lowest tan δ at 60 °C. In particular, as E60 and R60-T compounds have similar crosslink densities and crosslink structures, the effect of polymer dispersity on tan δ at 60 °C values can be easily compared. For R60-T, the tan δ at 60 °C was improved by 29% when compared to the E60 compound. Consequently, the narrower the dispersity of the base polymer, the lower the energy dissipation in the compound. Therefore, we believe that a compound for tire treads with excellent fuel efficiency can be manufactured using a polymer with a narrow dispersity.

#### 3.3.3. Mechanical Properties and Abrasion Resistance of ESBR and RAFT ESBR Carbon Black-Filled Compounds with a Controlled Cure system

**Figure 13:**
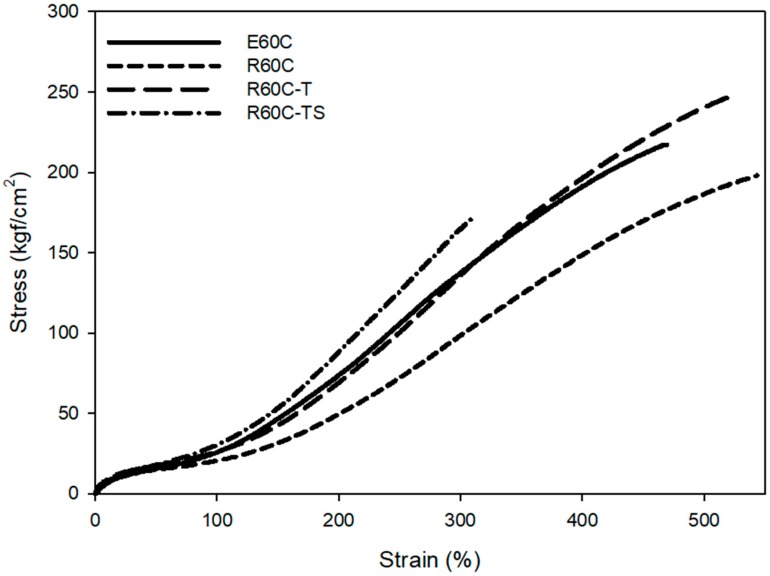
Mechanical properties of vulcanizates of emulsion styrene butadiene rubber (ESBR) and reversible addition-fragmentation radical transfer (RAFT) ESBR carbon black-filled compounds with a controlled cure system.

M_100%_ and M_300%_, which are closely related to the crosslink density, exhibited the same tendency as those of the crosslink density results in that E60 and R60-T compounds, having the same crosslink density, showed similar M_100%_ and M_300%_ values, as shown in Figure 13 and Table 11. The R60-TS compound with the highest crosslink density generated the highest M_300%_ and the lowest elongation-at-break values. In addition, there was a 9% improvement in the abrasion resistance for R60-T compounds with the same crosslink density as the E60 compound. Furthermore, RAFT ESBR compounds with low energy dissipation showed excellent abrasion resistance when compared to ESBR compounds. However, the highest crosslink density of the R60-TS compound resulted in a significant decrease in elongation due to the high crosslink density, resulting in a similar abrasion resistance to R60-T. This experiment confirmed that the abrasion resistance improved due to the narrow dispersity of RAFT ESBR.

## 4. Conclusions

In this study, a carbon black-filled compound was manufactured while using RAFT ESBR and ESBR, and our experiment confirmed that RAFT ESBR had a similar abrasion performance and excellent fuel efficiency characteristics when compared to that of ESBR, despite its lower crosslink density. The crosslink density and crosslink structure analysis confirmed that the low crosslink density of the RAFT ESBR compounds occurred because the trithiocarbonate groups consumed sulfur to form polysulfide structures, and these structures greatly reduced the chemical crosslink density of the compounds.

We regulated the di- and mono-crosslink structure in the RAFT ESBR compounds by increasing the amount of the crosslink accelerator, according to the results of the crosslink structure analysis. Through competing reactions between the trithiocarbonate groups in RAFT ESRB and the crosslink accelerator, the RAFT ESBR compounds retained the same crosslink density and crosslink structure as the conventional ESBR compounds. When comparing the compounds with similar crosslink densities and structures, the effect of the dispersity of the base polymer on the mechanical properties and the dynamic viscoelastic properties were analyzed. As a result, the RAFT ESBR showed 9% higher abrasion resistance and 29% higher fuel efficiency than the conventional ESBR. Therefore, the narrow dispersity of the base polymer can effectively reduce the energy dissipation in the tire tread compounds and greatly affect the abrasion resistance and fuel efficiency.

## Figures and Tables

**Table 1:** Experimental formulation for the reversible addition-fragmentation radical transfer emulsion styrene butadiene rubber (RAFT ESBR) polymerization; (Unit: g).

	Organic Compounds	ESBR	RAFT ESBR
Monomer	Styrene	112	
	Butadiene	288	
Chain transfer agent	*S,S*-dibenzyl trithiocarbonate	-	0.62
	*tert*-Dodecyl mercaptan	0.69	-
Solvent	3rd Distilled water	640	
Surfactant	Rosin soap	39.5	
	Fatty soap	100.1	
Catalyst	EDTA	0.1	
	FES	0.04	
	SFS	0.16	
Initiator	*p*-Menthane hydroperoxide	0.37	
Short stop agent	DEHA	*0.021*	

**Table 2:** Formulation of carbon black-filled compounds using emulsion styrene butadiene rubber (ESBR) and reversible addition-fragmentation radical transfer ESBR (RAFT ESBR); (Unit: phr).

	E40C	E60C	R40C	R60C
ESBR	100	100	-	-
RAFT ESBR	-	-	100	100
Carbon black (N330)	40	60	40	60
Zinc oxide	3	3	3	3
Stearic acid	1	1	1	1
Sulfur	1.8	1.8	1.8	1.8
TBBS	1.0	1.0	1.0	1.0

**Table 3:** Formulation of carbon black-filled compounds for increasing crosslink density; (Unit: phr).

	E60	R60	R60-T	R60-TS
ESBR	100		-	-
RAFT ESBR	-	100	100	100
Carbon black (N330)	60	60	60	60
Zinc oxide	3	3	3	3
Stearic acid	1	1	1	1
Sulfur	1.8	1.8	1.8	2.1
TBBS	1.0	1.0	1.3	1.3

**Table 4:** Mixing procedure for the carbon black-filled compounds.

Step	Time [mm:s]	Temp [°C]	RPM	Action
Carbon black masterbatch (CMB) mixing	00:00–00:40	110	20	add rubbers
	00:40–01:40		40	carbon black 1/2
	01:40–02:40		40	carbon black 1/2
	02:40–04:40		50	add ZnO, stearic acid
	04:40–05:00		50	ram up
	05:00–12:00		50	mixing
Final masterbatch (FMB) mixing	00:00–00:20	50	20	add MB
	00:20–01:00		40	add sulfur, TBBS
	01:00–02:00		40	mixing

**Table 5:** Characteristics of emulsion styrene butadiene rubber (ESBR) and reversible addition-fragmentation radical transfer ESBR (RAFT ESBR).

	Molecular Weight (M_w_)	Dispersity (Đ) (M_w_/M_n_)	Styrene Content (wt.%)	Vinyl Content (% in Butadiene)	Mooney Viscosity (ML_1+4_)
ESBR	540,000	4.1	23.5	20.7	52
RAFT ESBR	550,000	3.1	23.5	21.1	61
SSBR (5220M)	440,000	2.2	26.5	26.0	54

**Table 6:** Cure characteristics and crosslink densities of emulsion styrene butadiene rubber (ESBR) and reversible addition-fragmentation radical transfer (RAFT) ESBR carbon black-filled compounds.

	Unit	E60C	R60C
Mooney viscosity	ML_1+4_	68	75
t_10_	min:s	4:12	5:19
t_90_	min:s	10:47	11:14
T_min_	Nm	0.29	0.19
T_max_	Nm	2.05	1.60
T_max_ − T_min_	Nm	1.75	1.41
Total crosslink density	10^−4^ mol/g	0.8888	0.6449
Chemical crosslink density	10^−4^ mol/g	0.6456	0.4055
Filler-rubber interaction	10^−4^ mol/g	0.2432	0.2394
Poly crosslink density	10^−4^ mol/g	0.2744	0.2608
Ratio of poly crosslink density	%	30.9	40.4

**Table 7:** Viscoelastic properties of vulcanizates of emulsion styrene butadiene rubber (ESBR) and reversible addition-fragmentation radical transfer (RAFT) ESBR carbon black-filled compounds.

	ESBR	RAFT ESBR
*T*_g_ (°C)	−45	−45
Tan δ at *T*_g_	0.871	0.823
Tan δ at 0 °C	0.208	0.204
Tan δ at 60 °C	0.168	0.142

**Table 8:** Mechanical properties and abrasion resistance of vulcanizates of emulsion styrene butadiene rubber (ESBR) and reversible addition-fragmentation radical transfer (RAFT) ESBR carbon black-filled compounds.

	Unit	ESBR	RAFT ESBR
M_100%_	kgf/cm^2^	25	23
M_300%_	kgf/cm^2^	125	96
Elongation	%	406	510
DIN abrasion loss	mg	60	60

**Table 9:** Cure characteristics and crosslink densities of emulsion styrene butadiene rubber (ESBR) and reversible addition-fragmentation radical transfer (RAFT) ESBR carbon black-filled compounds with a controlled cure system.

	Unit	E60	R60	R60-T	R60-TS
Mooney viscosity	ML_1+4_	68	76	73	74
t_10_	min:s	4:08	4:13	4:16	4:10
t_90_	min:s	11:06	12:30	10:41	10:04
T_min_	Nm	0.27	0.20	0.20	0.20
T_max_	Nm	1.93	1.57	1.82	2.02
T_max_ − T_min_	Nm	1.66	1.37	1.62	1.82
Total crosslink density	10^−4^ mol/g	0.8672	0.6845	0.8630	0.9968
Poly crosslink density	10^−4^ mol/g	0.2724	0.2968	0.2782	0.2880
Ratio of poly crosslink density	%	31.4	43.4	32.2	28.9

**Table 10:** Viscoelastic properties of vulcanizates of emulsion styrene butadiene rubber (ESBR) and reversible addition-fragmentation radical transfer (RAFT) ESBR carbon black-filled compounds with a controlled cure system.

	Unit	E60	R60	R60-T	R60-TS
*T*_g_	°C	−45	−45	−43	−42
Tan δ at *T*_g_	-	0.871	0.815	0.810	0.802
Tan δ at 0 °C	-	0.200	0.202	0.196	0.203
Tan δ at 60 °C	-	0.164	0.141	0.116	0.103

**Table 11:** Mechanical properties and abrasion resistance of vulcanizates of emulsion styrene butadiene rubber (ESBR) and reversible addition-fragmentation radical transfer (RAFT) ESBR carbon black-filled compounds with a controlled cure system.

	Unit	E60	R60	R60-T	R60-TS
M_100%_	kgf/cm^2^	26	20	26	30
M_300%_	kgf/cm^2^	138	99	136	164
Elongation	%	470	540	520	310
DIN abrasion loss	mg	58	58	53	53
